# Analysis of Micro-Machining Process for External Thread of Micro Round Tube

**DOI:** 10.3390/ma14154327

**Published:** 2021-08-03

**Authors:** Tsung-Chia Chen, Jyun-Jie Lian, Cheng-Chi Wang

**Affiliations:** 1 Department of Mechanical Engineering, National Chin-Yi University of Technology, Taichung 41170, Taiwan; ctchen@ncut.edu.tw (T.-C.C.); monel9230@yahoo.com.tw (J.-J.L.); 2 Graduate Institute of Precision Manufacturing, National Chin-Yi University of Technology, Taichung 41170, Taiwan

**Keywords:** stainless steel, micro round tube, external threading, finite element

## Abstract

This study aims to analyze the stainless steel micro round tube external threading process for the influence of different outer threading pitches (0.25 mm, 0.4 mm) and outer diameters (Ø1.9, Ø1.94, Ø2). This study also analyzes the effects of different friction factors (0.1, 0.3, 0.5, 0.7, and 0.9) and different tube thicknesses (0.4, 0.45, 0.5, 0.55, and 0.6 mm) on the threading process. This study considers size effect to use corrected material parameters for the microtube to conduct the finite element analysis by DEFORM-3D software. The goal is to understand stainless steel (SUS304) micro round tube threading and the difference by using macro material parameter analysis. The historic forming data from the simulation and experiment of threading processing are presented, and the corresponding stress/strain distribution and thread shape are also calculated. The experiment results are compared to the simulation results to verify the reliability of this analysis method. The result shows that the torque/stress/strain obtained by the modified model is always lower than by Swift’s model. It means that the size effect can be considered to apply on the forming process and provided proper torque to form the external thread of the micro round tube, e.g., the maximum torque of the round die for M2 × 0.25 occurs over the fourth stroke. For the influence of the outer diameter of the micro round tube, the larger diameter induces the larger maximum torque on the round die for M2 × 0.4, but for the smaller pitch of M2 × 0.25, the larger maximum torque is not influenced by the diameter of the tube. When the pitch of the round die is increased, the torque, stress and strain are also increased relatively. As the friction factor and torque between the round die and tube increase, the stress and strain become lower. Changing the tube thickness will not significantly change the torque, the stress, and the strain. These results guide the simulation and experiment of optimized micro round tube threading development and design to reduce cost and increase product quality.

## 1. Introduction

Since its development in the 1950s, the screw industry has accumulated decades of manufacturing experience. In the last few decades, the industry has continued to transform and upgrade in response to global industry competition. The screw industry has not only crossed into high-end fields, such as the automotive, aerospace, and construction fields, and maintained its competitive advantage, but many screw and nut vendors have also moved towards higher-value fields, such as medical equipment, in recent years. Microforming is the process of using plastic deformation of material to produce millimeter-level or smaller parts in at least two dimensions. In industrial applications, this process is commonly used for forming processing under the 10 mm scope [1,2]. Microforming technology has the advantage of fast manufacturing speed and low cost and is suitable for mass production. Microforming processes provide a high-volume production for micro-parts and contribute a promising approach for manufacturing relevant micro-parts from metal materials. It is an important technology in micro manufacturing, and its technological core includes material, mold, testing and environment [3,4].

Huang [5] explored the impact of different screw head hammering and thread-rolling parameters on the forging force and metal flow patterns of small magnesium alloy LZ91 screws. Huang also used DEFORM-2D software to simulate and analyze the formability and production parameters of screw head hammering and thread-rolling technology for small-size screws. Chang [6] explored the development time for technology related to the manufacturing of magnesium alloy LZ91 screws. He used simulations to analyze the impact of various production parameters on formability, as well as conducted various production experiments. The experiment data and the simulation values were compared to verify the suitability of the model being analyzed. Li [7] used different tool geometric parameters and processing conditions to explore the impact of micro screw tapping on stainless steel sheet (SUS304) tapping. The tapping quality is determined based on the tapping torque and the tapped thread size. The Taguchi Method can only explore a single quality parameter and cannot solve multiple quality parameter problems. Thus, grey relational theory is used to obtain the best parameters for multiple optimal quality, which enabled the micro thread tapping tool to reach its maximum benefit. Kawka and Makinouchi [8] proposed the degenerated shell element and combined it with the static explicit finite element program. The program was used to compare different integration laws, such as full integration, integration by reduction, selective reduced integration, assumed strain field, and the stable matrix law.

In 2014, Chen [9] aimed to analyze the difference of stainless (SUS316L) microtubes in the flaring forming among dies with various semicone angles. Pawar and Joshi [10] found that when tapping small-diameter internal threads on titanium alloys, the wire taps often broke suddenly. Therefore, they set up an extensive experiment to study the tapping mechanism and the performance of axial vibration-assisted tapping (AVAT) and torsional vibration-assisted tapping (ATVAT) for titanium alloys. Oezkaya and Biermann [11] developed conventional tapping tools by using expensive investigational experiments. A method was proposed to predict relative torque in the design phase and thus save resources, energy, and costs. In 2019, Debnath, and Patowari [12] presented a method for three-dimensional complex geometry fabrication on the microscale where a typical two-axis wire-cut electric-discharge machine (WEDM) is used. The established process was further improved to fabricate screws of minimum possible dimension using the WEDM. Sharma et al. [13] developed novel maskless and masked techniques for fabricating both macro and micro thread profiles on cylindrical rotating shafts by using a wire electrochemical turning process (wire-ECTrg). For the research of testing microtubes’ characteristics [14,15], it was carried out on investigations into the influence of size effects on the forming behavior [16,17] and plastic deformation behavior [18,19] of metal materials by scaling down standard test methods.

The products analyzed by the microtube outer threading formation simulation analysis used in this study can be applied to micro parts such as electronic parts, watch/clock parts, miniature motors, medical equipment products, and general household electric appliances. The production process involves placing a stainless steel microtube in the pre-designed mold. The microtube is fixed on the lower die base, and the round die moves down the mold to the top of the microtube. As the round die lowers and turns, the microtube is tapped in the process and produces threading on the microtube. Thus, for this study, the microtube threading processing simulation is first analyzed. The threading processing of different pitches of round dies on microtubes is analyzed, as well as the effects of different friction factors and tube thicknesses. Experiments were conducted and the results were compared with the simulation to verify the accuracy of the simulation. Subsequently, the torque, forming history, and stress/strain were discussed.

## 2. Theory and Methods

### 2.1. Basic Assumptions

This study made several assumptions about the sheet forming and processing period.

- The material is assumed to be homogeneous.
- The round die and the lower die are perceived as rigid bodies.
- The formation equation of material includes the isotropic strain hardening effect.
- The strain rate of material considers elasticity and plastic strain rate.
- During formation, the temperature and internal stress of material are not considered.
- The material follows Hooke’s Law in the elastic area.
- The material follows the von Mises criterion; after the plastic deformation, the material follows the Prandtl–Reuss plastic flow law.

### 2.2. Size Effect-Modified Micro Elastic-Plastic Material Model

Because of the size effect during the microforming process, there is a large difference between the finite element analysis and the actual product, making the traditional material model unsuitable. Thus, a new material model that considers the size effect must be constructed. SUS304 was used in the tensile experiment to determine the impact of the size effect on microtubes and construct a new micro elastic-plastic material model. The new model is compared with the traditional material model to evaluate the difference. The following Swift material model was used:

(1) $$\bar{\sigma }=K{\left(\varepsilon_{0}+\bar{\varepsilon_{p}}\right)}^{n}$$

where $\bar{\sigma }$ and $\bar{\varepsilon_{p}}$ represent the equivalent stress and strain, respectively, and *K*, *n* and $\varepsilon_{0}$ are constants for a particular material, determined usually in uniaxial tension tests. When the tube thickness is less than 1.0 mm, the size effect becomes apparent. However, when the tube thickness is greater than 1.0 mm, the size effect can be omitted. The material thickness used in this study is 0.5 mm. Therefore, the original material model was corrected as shown below:

(2) $$\bar{\sigma }(t,\bar{\varepsilon })=aKe^{bt}{\left(\varepsilon_{0}+\bar{\varepsilon_{p}}\right)}^{n(ce^{dt}-1)}$$

where *a, b, c, d* are the correction proportion and *t* is the tube thickness. The correction value was obtained by Fang Liu [20], and the substituted equation is as follows:

(3) $$\bar{\sigma }(t,\bar{\varepsilon })=0.73667Ke^{0.3152t}{\left(\varepsilon_{0}+\bar{\varepsilon_{p}}\right)}^{n(1.0106e^{-0.01029t}-1)}$$

In this study, SUS 304 stainless steel is used to be the experimental materials and the material parameters are shown in Table 1. Where *E* is the Young’s modulus, *σ_y_* is the yielding stress.

This study conducted finite element analysis on the corrected material model as shown in Equation (3) and compared the difference between the two models with the experiment results.

## 3. Numerical Analysis

**Figure 1:**
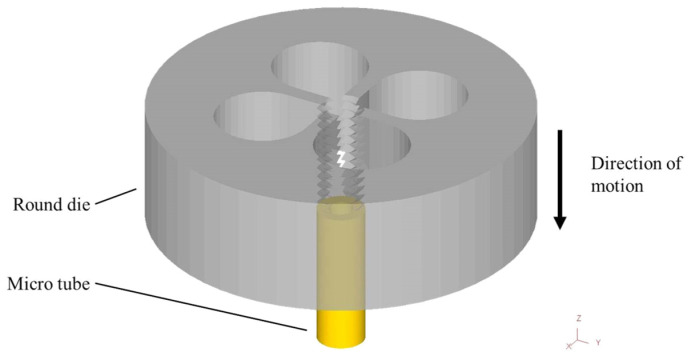
Simulation setup of round die and microtube.

This study used shape function derived from the 3D tetrahedron four-node element and coupled the function to the rigidity matrix. Computer-aided design (CAD) software was used for the computer processing to partition the established tube exterior into grids and to convert the information into data files. DEFORM-3D finite element analysis software (V10) was used to simulate two round dies (M2 × 0.25 and M2 × 0.4). The influence of temperature was not considered. Microtubes were assumed to be plastic bodies, and the materials were assumed to be homogenous and isotropic. The friction between the round die and the microtube was assumed to be constant shear friction. The operating modes of the round die and microtubes are displayed in Figure 1.

Shape function coupling as derived from a 3D tetrahedral four-node element was applied to the stiffness matrix, and CAD was used to create a mesh segmentation of the tube profile that was then transferred into a data file and input to the finite element program for use in simulations. The simulation results were output to the CAD program for interpretation and analysis. The software could display a formation diagram and the stress–strain distribution. The process parameters of screw pitch, friction factor, and tube thickness affected the thread processing of the microtube, and the relation between torque and stroke and the distributions of maximum principal stress and maximum principal strain were analyzed.

When DEFORM-3D is used to simulate thread processing, the number of grids affects the shape of the thread. The number of grids is larger, the time required for analysis and calculation is longer, and the data required to store analysis and calculation is quite large. In order to make the simulation of thread processing more efficient, the number of grids is set to 60,000 for analysis. The simulation parameters are shown in Table 2.

The material model and the difference with the experiment result are explored during the threading process. The round die comes into contact with the tube surface as the threading begins, so the nodes that engage and disengage with the round die should be defined. The nodes are divided into contact nodes and free nodes. Non-contact nodes are defined as free nodes. Global space coordinates (X, Y, and Z) are used. For the node that comes in contact with the round die, the right-hand rule was used to define the current local coordinate position (ξ,η,ζ). The simulation process is shown as following:

(1). Save the product mold model designed in CAD in the STL file format and import it into the DEFORM-3D software.
(2). Use the meshing tool provided by DEFORM-3D to mesh the workpiece.
(3). Set the boundary conditions of the workpiece.
(4). Establish properties of mold and workpiece, and set corresponding process parameters (contact properties, friction factors, etc.).
(5). Adjust the corresponding position between mold and workpiece and observe the relative movement between them to ensure the correctness of the mold movement.
(6). Set the analysis and calculation parameters and analyze them through the DEFORM-3D simulation engine.
(7). Pass the analysis results through the DEFORM-3D post-processor and display the numerical simulation results in the form of distribution graphs or animations.

## 4. Experiment Verification

### 4.1. Torque Comparison in the Simulation and the Experiment

**Figure 2:**
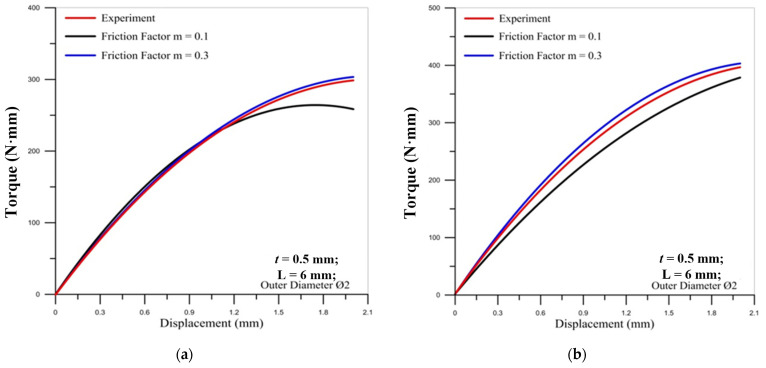
Comparison of the torques by simulation and experiment: (**a**) M2 × 0.25; (**b**) M2 × 0.4.

Figure 2 shows the comparison of torques between the simulation and experiment. To determine the friction factor set for the simulation, this study first assumed that the friction factor was 0.1 and 0.3 to compare with the experiment value and make the verification. When friction factor m = 0.3, the simulated torque curve and the experimental curve were close in the figure. Thus, this study used a friction factor of m = 0.3 for the simulation value to analyze the microtube threading forming process.

### 4.2. Comparison of Thread Shape in the Simulation and Experiment

**Figure 3:**
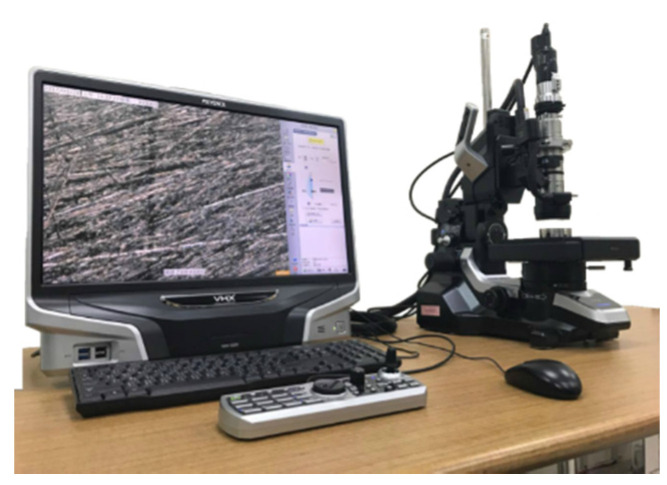
Optical microscope (KEYENCE VHX-5000, Osaka, Japan).

**Figure 4:**
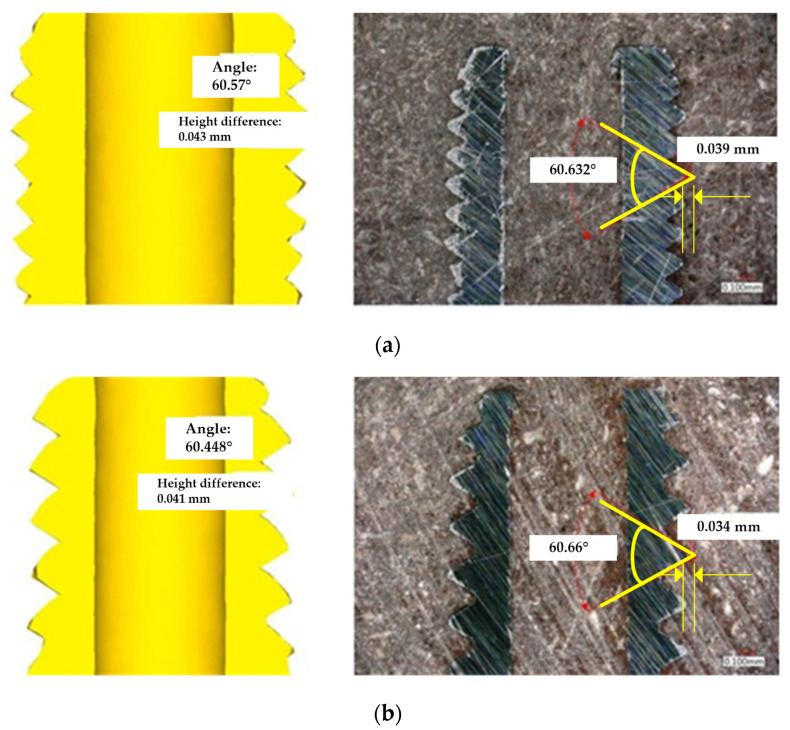
Comparison of simulated and experiment thread shape at 100× magnification: (**a**) M2 × 0.25; (**b**) M2 × 0.4.

To verify the accuracy of the simulation and experiment in this study, an optical microscope was used to measure the thread shape angle and thread shape height difference of the finished product shown in Figure 3. This is to explore the difference between the simulation and the experiment. Figure 4 shows the comparison of simulated and experiment thread shape. The figure shows that the thread shape angle of the M2 × 0.25 was 60.57° by simulation shown in the left of Figure 4a, and the experimental thread shape angle was 60.632° shown in the right of Figure 4a. The error rate of angle is approximately 0.1%. The thread shape height difference by simulation was 0.042 mm and the experimental thread shape height difference was 0.039 mm, which is an error rate of approximately 7.69%. For the M2 × 0.4 case, the thread shape angle was 60.448° by simulation while the experimental thread shape angle was 60.660°, which is an error rate of approximately 0.34%. The simulation’s thread shape height difference was 0.037 mm and the experiment’s thread shape height difference was 0.034 mm, which is an error rate of approximately 8.82%.

## 5. Results and Discussion

This section is divided into three sub-sections, and the parameters involved are pitch, friction factor, and tube thickness.

### 5.1. Forming History

**Figure 5:**
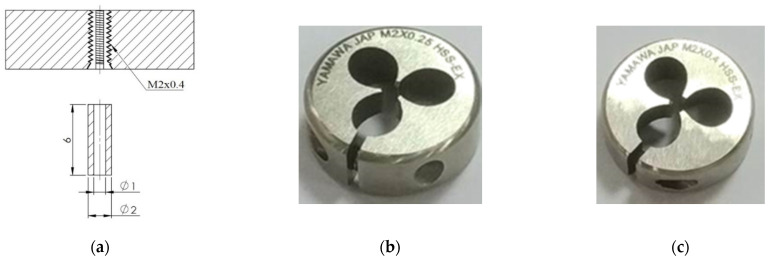
Illustration of the (**a**) round die and the tube size: (**b**) M2 × 0.25; (**c**) M2 × 0.4.

**Figure 6:**
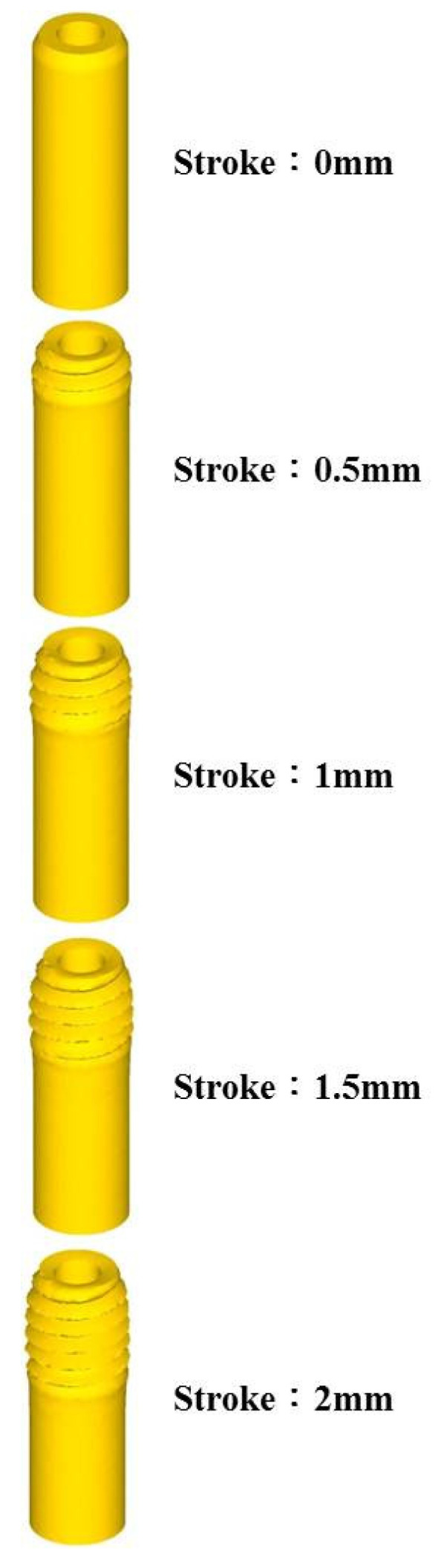
Forming history.

For the simulation, the pitch values used were 0.25 and 0.4 mm. The friction factor values used were 0.1, 0.3, 0.5, 0.7, and 0.9. The tube thicknesses used were 0.4, 0.45, 0.5, 0.55, and 0.6 mm. Figure 5 shows the round die and the position of the raw material. Figure 6 is the geometric forming of the five stages of the microtube threading process.

### 5.2. Torque Comparison

**Figure 7:**
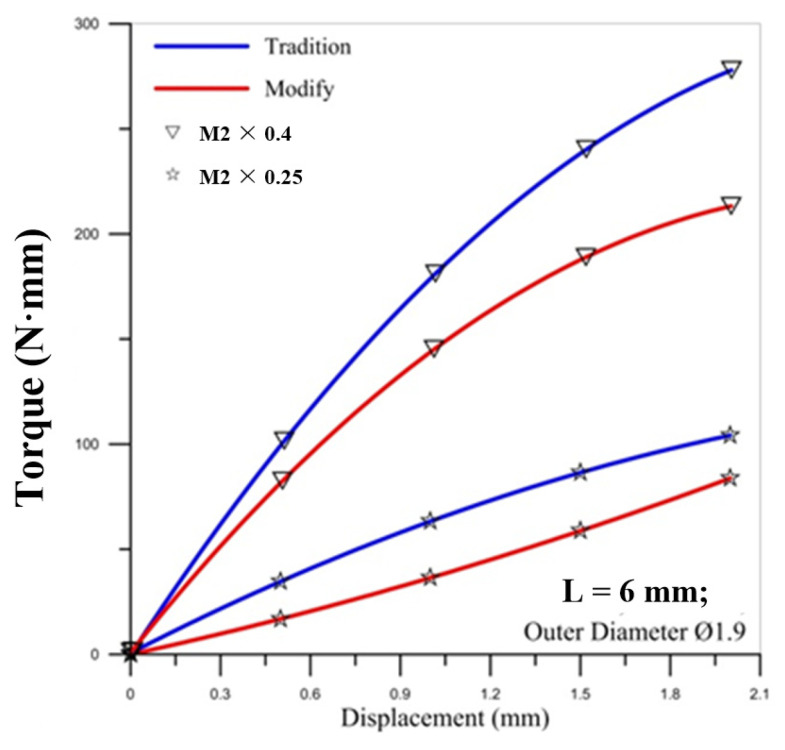
Correlation between torque of round die and displacement of stroke with two pitches of round die (outer diameter Ø1.9).

**Figure 8:**
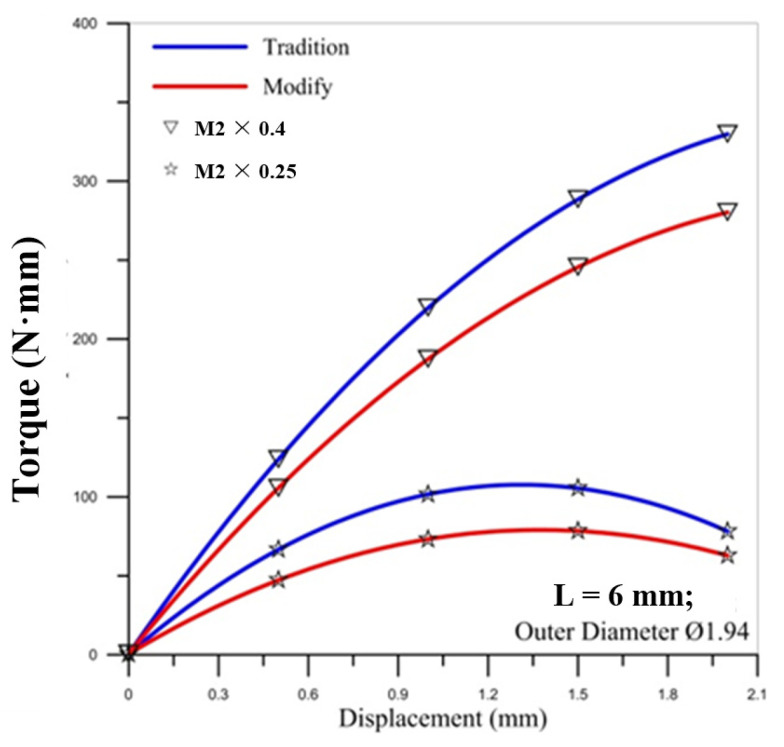
Correlation between torque of round die and displacement of stroke with two pitches of round die (outer diameter Ø1.94).

**Figure 9:**
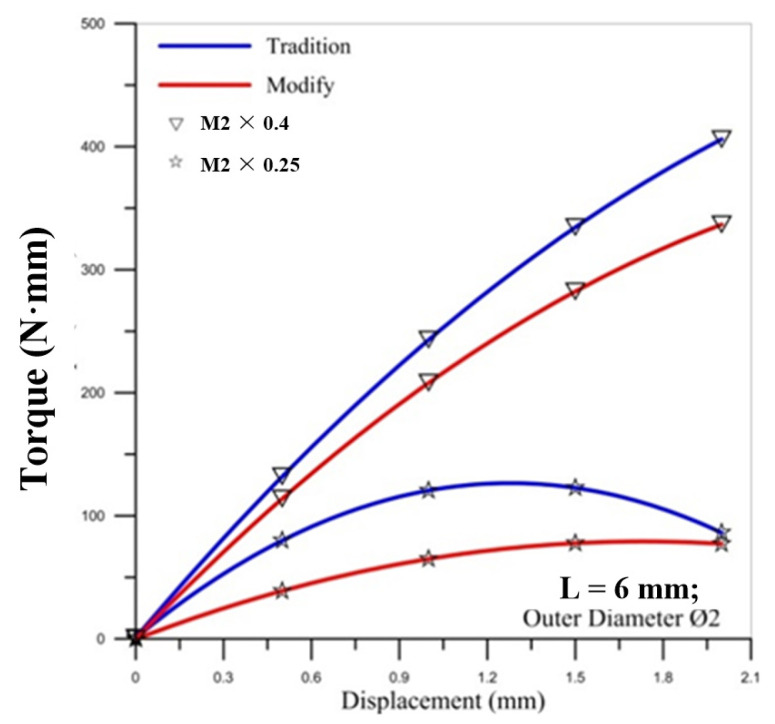
Correlation between torque of round die and displacement of stroke with two pitches of round die (outer diameter Ø2).

Figure 7, Figure 8 and Figure 9 show the correlation between the torque of the round die and the displacement of stroke with two pitches of the round die. The torque of the round die increases rapidly when in contact with the microtube. As the round die comes in contact with the microtube, the tapping process increases the torque of the round die and the torque becomes higher as the pitch becomes larger. The red curve of corrected modified torque using Fang Liu’s modified model is always lower than the blue curve of uncorrected traditional torque using Swift’s model. In Figure 8 and Figure 9, the maximum torque of the round die for the modified model of M2 × 0.25 occurs over the fourth stroke. The maximum value of torque for the larger pitch of M2 × 0.4 is triple that of M2 × 0.25.

For the influence of the outer diameter of the micro round tube, the larger diameter induces the larger maximum torque on the round die for the larger pitch of M2 × 0.4, but the maximum values of torque for the smaller pitch of M2 × 0.25 are not influenced by the diameter of the tube.

**Figure 10:**
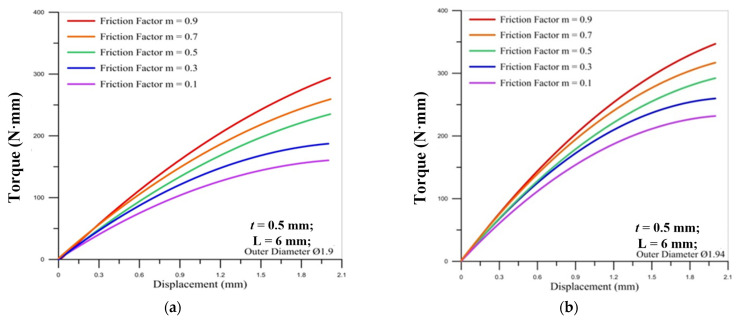

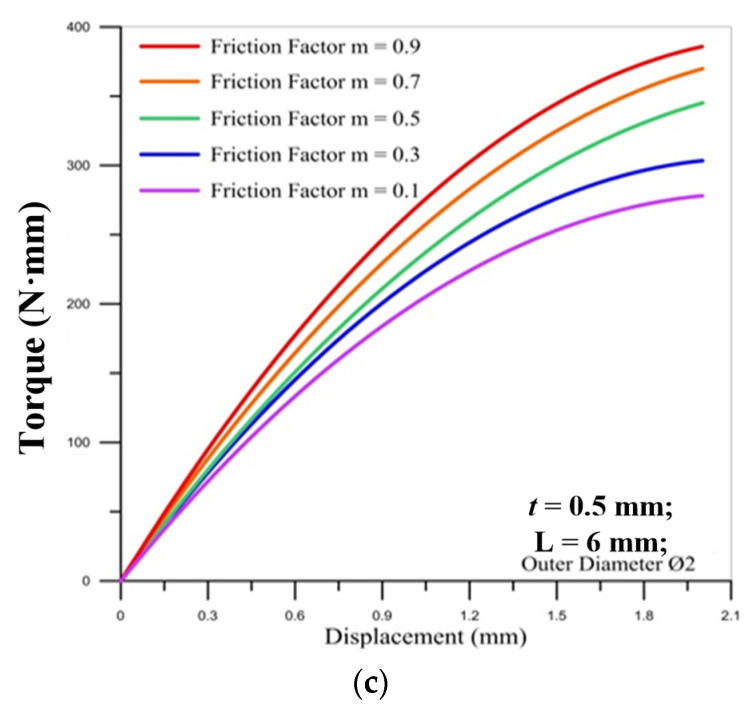
Correlation between torque of round die (M2 × 0.25) and displacement of stroke under different friction factors for outer diameter of micro round tube: (**a**) Ø1.9; (**b**) Ø1.94; (**c**) Ø2.

**Figure 11:**
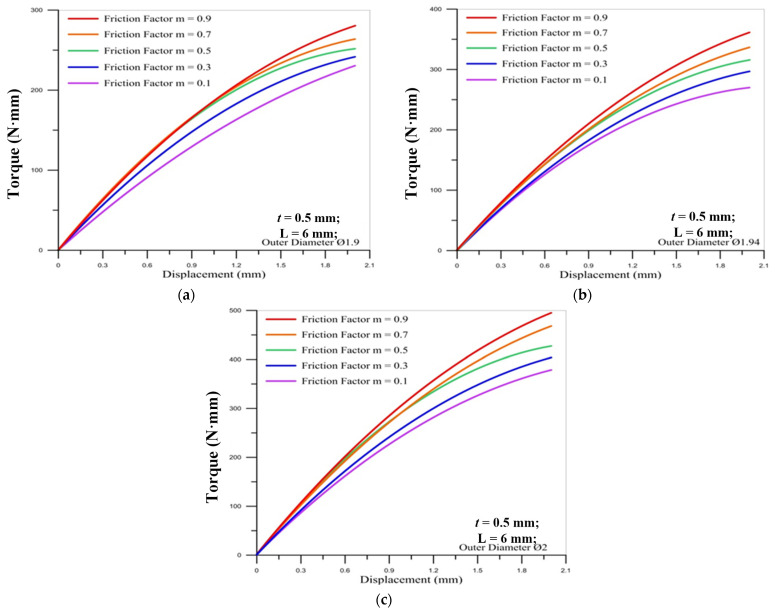
Correlation between torque of round die (M2 × 0.4) and displacement of stroke under different friction factors for outer diameter of micro round tube: (**a**) Ø1.9; (**b**) Ø1.94; (**c**) Ø2.

Figure 10 and Figure 11 show the correlation between the torque of the round die and the displacement of stroke under different friction factors for three sizes of the micro round tube outer diameter. It is found that the torque of the round die increases as the friction factor becomes larger. When the thread pitch is increased, the thread height is larger, so the torque required during processing is increased, and when the friction factor is larger, it will cause higher processing resistance. The maximum value of torque occurs over the final stroke for two different pitches of die. Moreover, the lager pitch (M2 × 0.4) induces the larger maximum values of torque on the round die than smaller pitch (M2 × 0.25) during the strokes of processing. For the influence of the outer diameter of the tube, the larger diameter causes the larger maximum torque on the round die.

**Figure 12:**
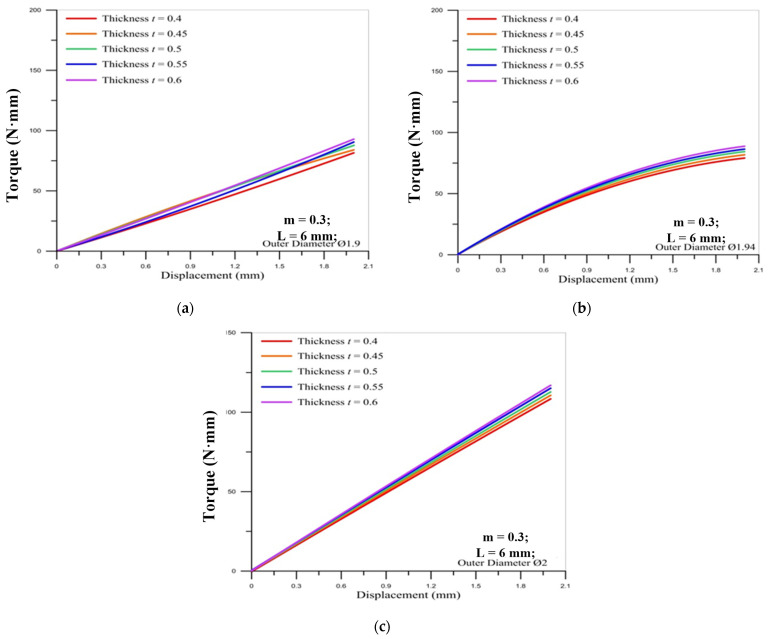
Correlation between torque of round die (M2 × 0.25) and displacement of stroke under different tube thicknesses for outer diameter of micro round tube: (**a**) Ø1.9; (**b**) Ø1.94; (**c**) Ø2.

**Figure 13:**
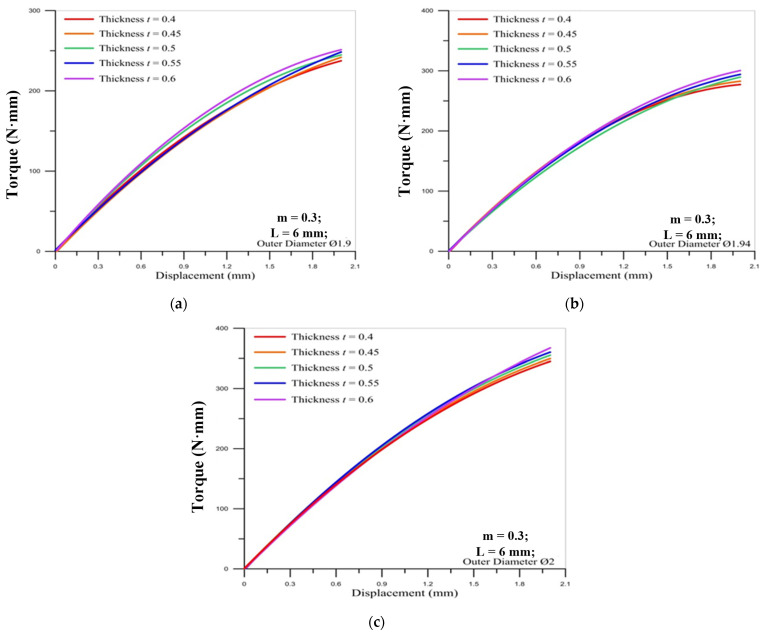
Correlation between torque of round die (M2 × 0.4) and displacement of stroke under different tube thicknesses for outer diameter of micro round tube: (**a**) Ø1.9; (**b**) Ø1.94; (**c**) Ø2.

Figure 12 and Figure 13 show the correlation between the torque of the round die and the displacement of stroke under different tube thicknesses for three sizes of tube outer diameter. The results reveal that the tube thickness in the microtube threading process does not significantly impact the torque of the round die. The maximum value of torque occurs over the final stroke for two different pitches of die. Moreover, the lager pitch (M2 × 0.4) induces the larger maximum values of torque on the round die than smaller pitch (M2 × 0.25).

### 5.3. Differences in Stress and Strain

**Figure 14:**
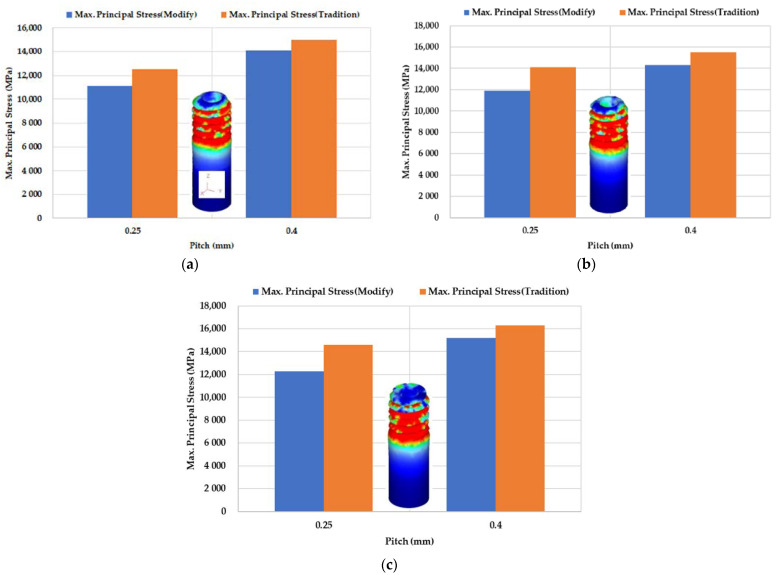
Correlation between pitch and maximum principal stress (M2, L = 6 mm) by Swift model (traditional uncorrected-orange column) and Fang Liu’s model (modified corrected-blue column) with outer diameter: (**a**) Ø1.9; (**b**) Ø1.94; (**c**) Ø2.

Figure 14 show the correlation between pitch and maximum principal stress. The maximum principal stress becomes higher as the pitch becomes larger. Figure 14a shows the M2 round die tapping a tube with an outer diameter of Ø1.9. After corrections, when the pitch was 0.25 mm, the maximum principal stress was 11,100 MPa. When the pitch was 0.4 mm, the maximum principal stress was 14,100 MPa. Figure 14b shows the M2 round die tapping a tube with an outer diameter of Ø1.94. After corrections, when the pitch was 0.25 mm, the maximum principal stress was 11,900 MPa. When the pitch was 0.4 mm, the maximum principal stress was 14,300 MPa. Figure 14c shows the M2 round die tapping a tube with an outer diameter of Ø2. After corrections, when the pitch was 0.25 mm, the maximum principal stress was 12,300 MPa. When the pitch was 0.4 mm, the maximum principal stress was 15,200 MPa. The corrected stress becomes smaller than the uncorrected stress.

**Figure 15:**
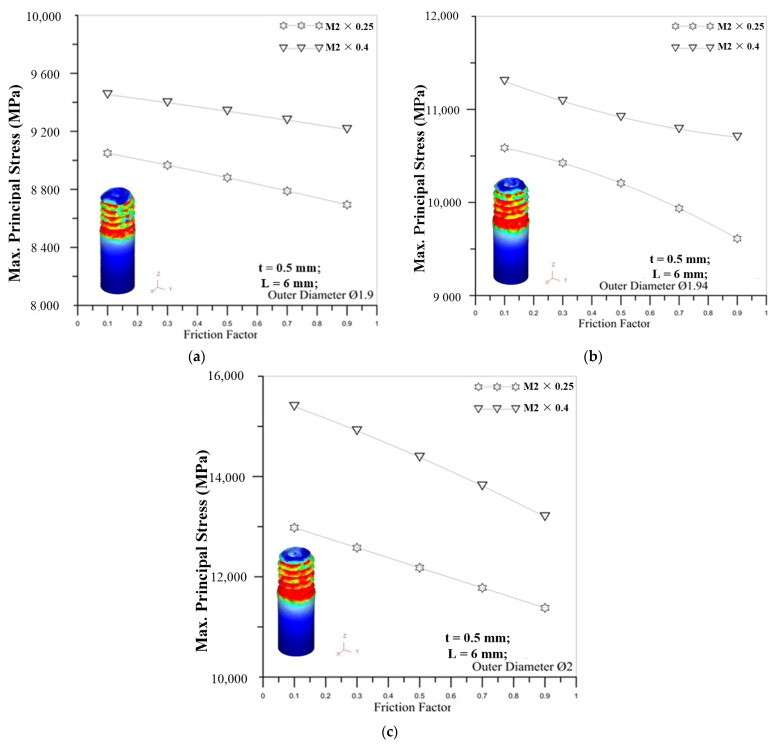
Correlation between friction factors and maximum principal stress for M2 × 0.25 and M2 × 0.4 with outer diameter of micro round tube: (**a**) Ø1.9; (**b**) Ø1.94; (**c**) Ø2.

Figure 15 shows the correlation between friction factors and maximum principal stress. The figures show that the maximum principal stress becomes smaller as the friction factor increases. It is due to the fact that the resistance between the round die and the surface of the microtube becomes larger during the forming process, making the external thread of the round tube less easily deformed, so the stress is relatively small. For the influence of the outer diameter of the tube, the larger diameter of the round tube induces the larger maximum principal stress for different friction factors.

**Figure 16:**
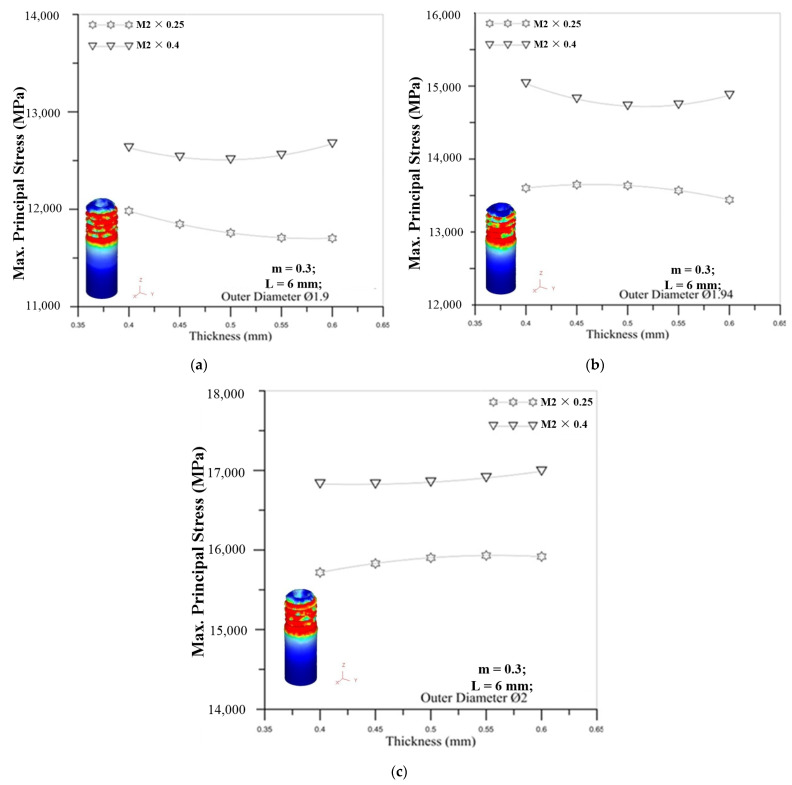
The correlation between tube thickness and maximum principal stress for M2 × 0.25 and M2 × 0.4 with outer diameter of micro round tube: (**a**) Ø1.9; (**b**) Ø1.94; (**c**) Ø2.

Figure 16 show the correlation between tube thickness and maximum principal stress. From the experiments and simulations, the results both reveal that an increase in tube thickness does not have a significant impact on maximum principal stress. For the influence of the outer diameter of the tube, the larger diameter of the round tube induces the larger maximum principal stress for different tube thicknesses. It can be seen that the influence of the diameter of the micro round tube on the maximum stress value is more important than the thickness of the tube.

**Figure 17:**
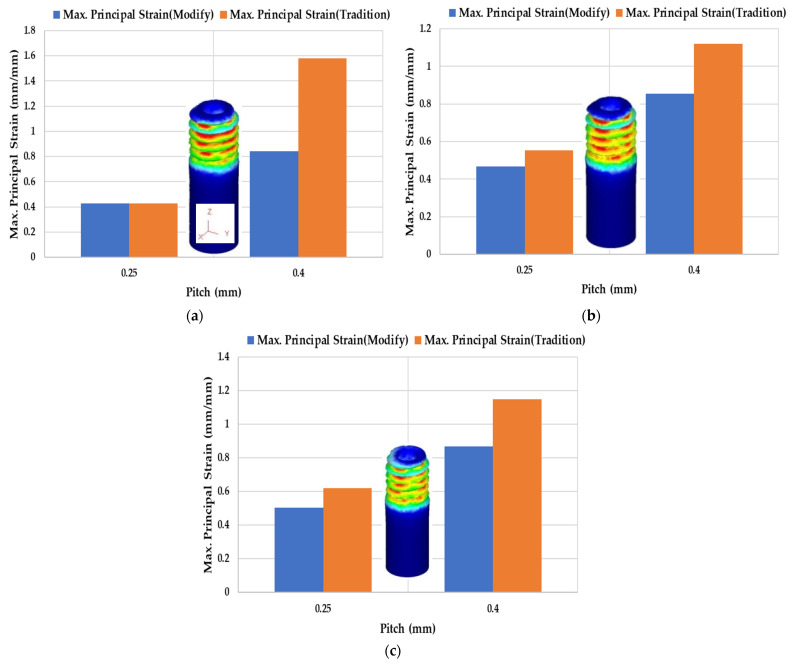
The correlation between pitch and maximum principal strain (M2) by Swift model (traditional uncorrected-blue curve) and Fang Liu’s model (modified corrected-red curve) with outer diameter: (**a**) Ø1.9; (**b**) Ø1.94; (**c**) Ø2.

As shown in Figure 17, the maximum principal strain is concentrated at the thread and becomes higher as the pitch increases. Figure 17a shows the M2 round die tapping a tube with an outer diameter of Ø1.9. For the modified material model, when the pitch was 0.25 mm, the maximum principal strain was 0.426 mm/mm. The difference of the strain values of these two model approaches 0. When the pitch was 0.4 mm, the maximum principal strains of the traditional uncorrected model and modified corrected model were 1.58 and 0.844 mm/mm, respectively. It is found that the strain obtained by the Swift model is twice by Fang Liu’s model for M2 × 0.4.

In Figure 17b, when the pitch was 0.25 mm, the maximum principal strain of the modified model was 0.466 mm/mm and less than the value 0.554 mm/mm of the traditional model. When the pitch was 0.4 mm, the maximum principal strain of modified model was increased to 0.855 mm/mm and still less than the uncorrected traditional model.

Figure 17c shows that the maximum principal strain of the modified model was 0.504 mm/mm as the pitch was 0.25 mm. Furthermore, when the pitch was 0.4 mm, the maximum principal strain of the modified model was 0.868 mm/mm. The corrected strain becomes smaller than the uncorrected strain.

**Figure 18:**
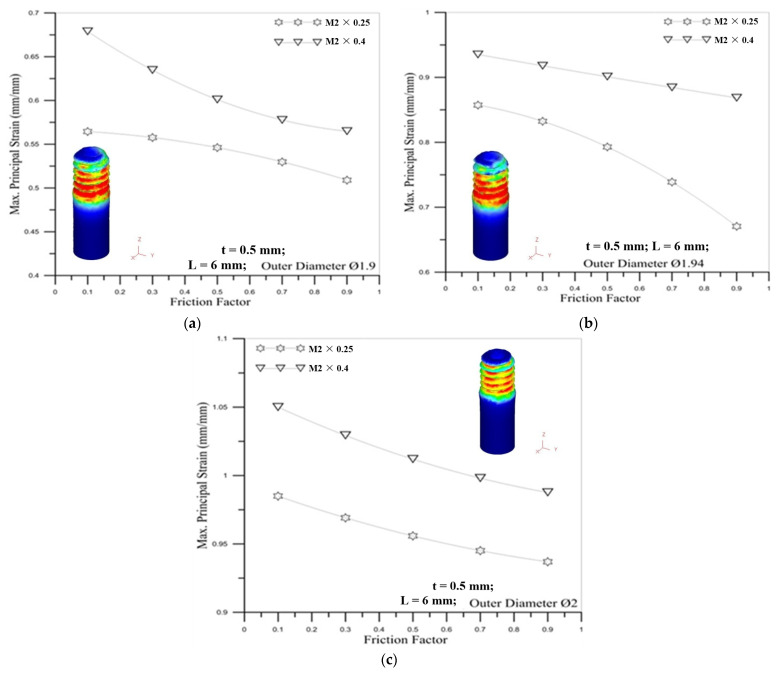
Correlation between friction factor and maximum principal strain for M2 × 0.25 and M2 × 0.4 with outer diameter of micro round tube: (**a**) Ø1.9; (**b**) Ø1.94; (**c**) Ø2.

Figure 18 shows the correlation between friction factor and maximum principal strain. The figures show that the maximum principal strain becomes smaller as the friction factor increases. When the friction factor is larger, the resistance between the round die and the microtube becomes larger during the forming process, making the external thread of the round tube less easily deformed, so the strain is relatively small. For the influence of the outer diameter of the tube, the larger diameter of the round tube induces the larger maximum principal strain for different friction factors.

**Figure 19:**
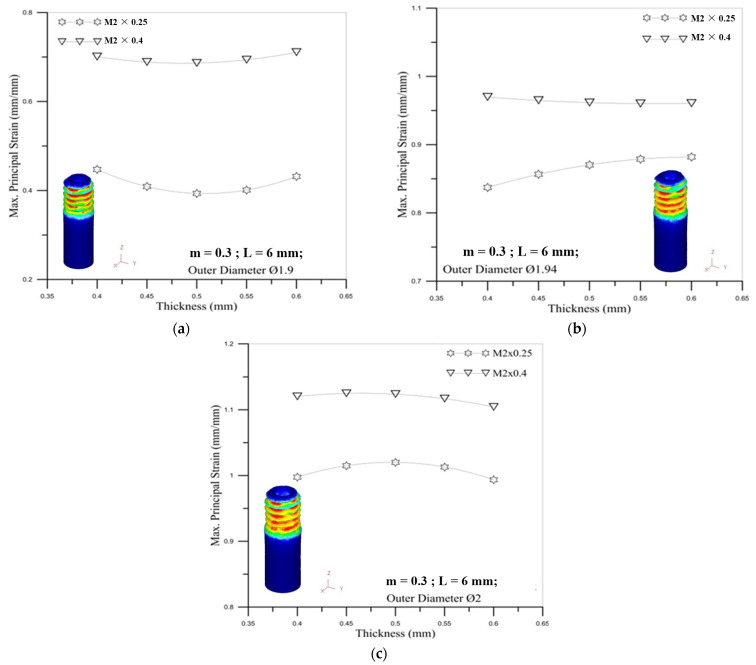
Correlation between tube thickness and maximum principal strain for M2 × 0.25 and M2 × 0.4 with outer diameter of micro round tube: (**a**) Ø1.9; (**b**) Ø1.94; (**c**) Ø2.

Figure 19 reveals the correlation between tube thickness and maximum principal strain. The figures show that tube thickness does not have a significant impact on the maximum principal strain. For the influence of the outer diameter of the tube, the larger diameter of the round tube induces the larger maximum principal stress for different tube thicknesses. It can be seen that the influence of the diameter of the micro round tube on the maximum stress value is more important than the thickness of the tube.

## 6. Conclusions

This study focuses on the micro round tube external threading process and analyzes different effects, including threading pitch, friction factor, outer diameter, and thickness of the round tube to cause the influence on the product. For the microtube, the size effect was considered to construct a micro elastic-plastic material model to correct the impact of downsizing on the micro round tube. This corrected model from simulations and experiments were compared to verify that it can be effectively applied to the entire microtube threading formation process. The results reveal that DEFORM-3D finite element analysis software can accurately analyze the complete deformation process in the micro round tube threading forming process. It is found that the maximum value of torque for the larger pitch of the round die (M2 × 0.4) is triple the pitch of M2 × 0.25 and the torque of the round die increases as the friction factor becomes larger. When the thread pitch is increased, the thread height is larger, so the torque required during processing is increased, and the maximum principal stress and maximum principal strain are both increased as well. Due to the higher pitch-induced higher torque required, the stress and strain are also increased. However, as the friction factor is larger, it will cause higher processing resistance, and the maximum principal stress and the maximum principal strain will be decreased. The reason for this is that the resistance between the round die and the microtube becomes larger as the friction factor increases during the forming process, making the outer thread of the round tube less easily deformed, so the stress and strain are relatively small. Moreover, when we change the tube thickness by experiments and simulation, the results both reveal that it would not cause an obvious impact on the torque of the round die, maximum principal stress, and maximum principal strain, respectively. It can also be seen that the influence of the diameter of the micro round tube on the maximum stress/strain is more important than the thickness of the tube. These results guide the simulation and experiment of optimized microtube threading development and design to reduce cost and increase product quality.

## Figures and Tables

**Table 1:** Stainless steel microtube material parameters.

Microtube Thickness	*E* (GPa)	*σ_y_* (MPa)	*K* (MPa)	*n*	$\varepsilon_{0}$
t = 0.5 mm	207	316	1568.7	0.5791	0.077

**Table 2:** Setup of simulation parameters.

Material	Outer Diameter	Inner Diameter	Tube Length	Constant Shear Friction	Temperature
SUS304	2 mm	1 mm	6 mm	0.3	25 °C
